# Pregnancy-Related Attack in Neuromyelitis Optica Spectrum Disorder With AQP4-IgG: A Single-Center Study and Meta-Analysis

**DOI:** 10.3389/fimmu.2021.800666

**Published:** 2022-01-05

**Authors:** Shuwen Deng, Qiang Lei, Wei Lu

**Affiliations:** Department of Neurology, The Second Xiangya Hospital, Central South University, Changsha, China

**Keywords:** pregnancy, aquaporin-4, neuromyelitis optica spectrum disorder, neutrophil-to-lymphocyte ratio, autoimmune diseases

## Abstract

**Objective:**

This study aimed to investigate the demographic characteristic of pregnancy-related attacks (PRAs) in neuromyelitis optica spectrum disorder (NMOSD). In addition, we investigated the predictors of PRAs as well as the effect of immunosuppressive (IS) therapy in patients with pregnancy-related NMOSD.

**Method:**

We retrospectively analyzed data on clinical and diagnostic characteristics, therapeutic management, and pregnancy outcomes for PRAs in AQP4-IgG-positive NMOSD patients admitted to the Second Xiangya Hospital of Central South University. Moreover, we searched the literature (without any temporal restriction) to identify all such similar cohorts and performed a meta-analysis to evaluate the effectiveness and safety of IS therapy on NMOSD patients with PRAs.

**Result:**

We collected clinical data on 117 women with AQP4 antibody-positive NMOSD; we ultimately included 33 patients (34 pregnancies). Ten patients were relapse-free during pregnancy, and 23 (69.7%) had PRA; attacks were most common during the first trimester of the postpartum period. Maintenance of IS treatment during pregnancy was found to greatly reduce PRAs in patients with NMOSD. PRAs were associated with a higher neutrophil-to-lymphocyte ratio (NLR) at relapse during pregnancy and shorter time interval between the last relapse and conception. The meta-analysis suggested that maintenance of IS treatment during pregnancy can significantly reduce the RR of NMOSD (95%CI=0.35-0.62; z=5.18, *p*<0.0001) and had no adverse effect on the miscarriage rate. However, the unhealthy newborn occurrence among those receiving IS treatment was 3.73 times higher than that of those not receiving treatment during pregnancy (95%CI=1.40–9.91; z=2.64, *p*=0.008).

**Conclusion:**

Our study results demonstrates that pregnancy can induce the onset or relapse of attacks in NMOSD patients. The increased NLR value and disease activity may be a predictor for PRAs in patients with NMOSD. Moreover, administration of IS treatment during pregnancy can reduce the relapse rate. However, the dosage of drugs and risks of adverse effects to the fetus need to be considered. Future prospective studies with larger sample sizes are needed to confirm and extend our findings.

## 1 Introduction

Neuromyelitis optica spectrum disorder (NMOSD) is a serious, recurrent antibody-mediated inflammatory demyelination disorder that is mainly characterized by optic neuritis and transverse myelitis; however, it can also affect other areas of the central nervous system (e.g., the brainstem and hypothalamus). This disease causes blindness, paralysis, cognitive impairment (1). Several studies have suggested increased relapse rates and a higher risk of disability in patients with NMOSD occurring during pregnancy and the postpartum period (2, 3). Because NMOSD relapse during pregnancy has adverse effects on both the pregnant woman and the fetus, researchers have suggested the maintained administration of immunosuppressive (IS) treatment for NMOSD following pregnancy (4). However, there have been few cohort studies on the curative effect of IS treatment with respect to prognosis and relapse rates in pregnant patients with seropositive NMOSD (5, 6). Until now, cohort studies have been limited by their small number of cases (7). Delgado et al. were the first to retrospectively analyze the obstetric outcome of patients with NMOSD and AQP4-IgG positivity in Latin America, and only one pregnancy was reported after the first manifestation of NMOSD among 50 pregnancies as there was a decrease in the desire for motherhood after the diagnosis of NMOSD (8). Therefore, it is difficult to interpret the results and make clear conclusions as to whether IS treatment during pregnancy can prevent disease relapse.

Aquaporin-4 IgG is a specific diagnostic and serological marker of NMOSD (9) that binds to AQP4 in astrocyte feet and regulates complement recruitment and activation, thereby inducing complement-dependent cytotoxicity and leading to astrocyte damage (10). Additionally, effector neuroinflammatory cells (including eosinophils, neutrophils, and macrophages attracted by proinflammatory cytokines) lead to astrocytic death, secondary oligodendrocyte injury, and axonal injury (11). Substantial sex hormone shifts occur during gestation and puerperium in NMOSD, and this can alter the amount and function of AQP4 IgG and also the activation of effector neuroinflammatory cells (3). Therefore, pregnancy is a risk factor for relapse in NMOSD patients (12). The neutrophil-to-lymphocyte ratio (NLR) is a novel inflammatory marker and an easily obtained parameter (13). The NLR has been used to predict prognosis and disease activity in many neurologic demyelinating diseases, including NMOSD (14) and multiple sclerosis (15), and in other systemic immunologic diseases, such as systemic lupus erythematosus (16), Sjogren’s syndrome (SS) (17), and Behçet’s disease (18). There are no previous studies on the association between relapse rate (RR) and the NLR in patients with pregnancy-related NMOSD.

Therefore, the primary objective of this cohort study was to summarize the demographic and clinical characteristics of patients with pregnancy-related NMOSD attacks. We analyzed the risk factors for pregnancy-related attacks (PRAs), including IS treatment and NLR. In addition, we performed a meta-analysis to investigate the efficacy of IS treatments in reducing the RR during pregnancy in women with NMOSD.

## 2 Methods

### 2.1 Meta Analysis

#### 2.1.1 Data Sources and Searches

**Figure 1:**
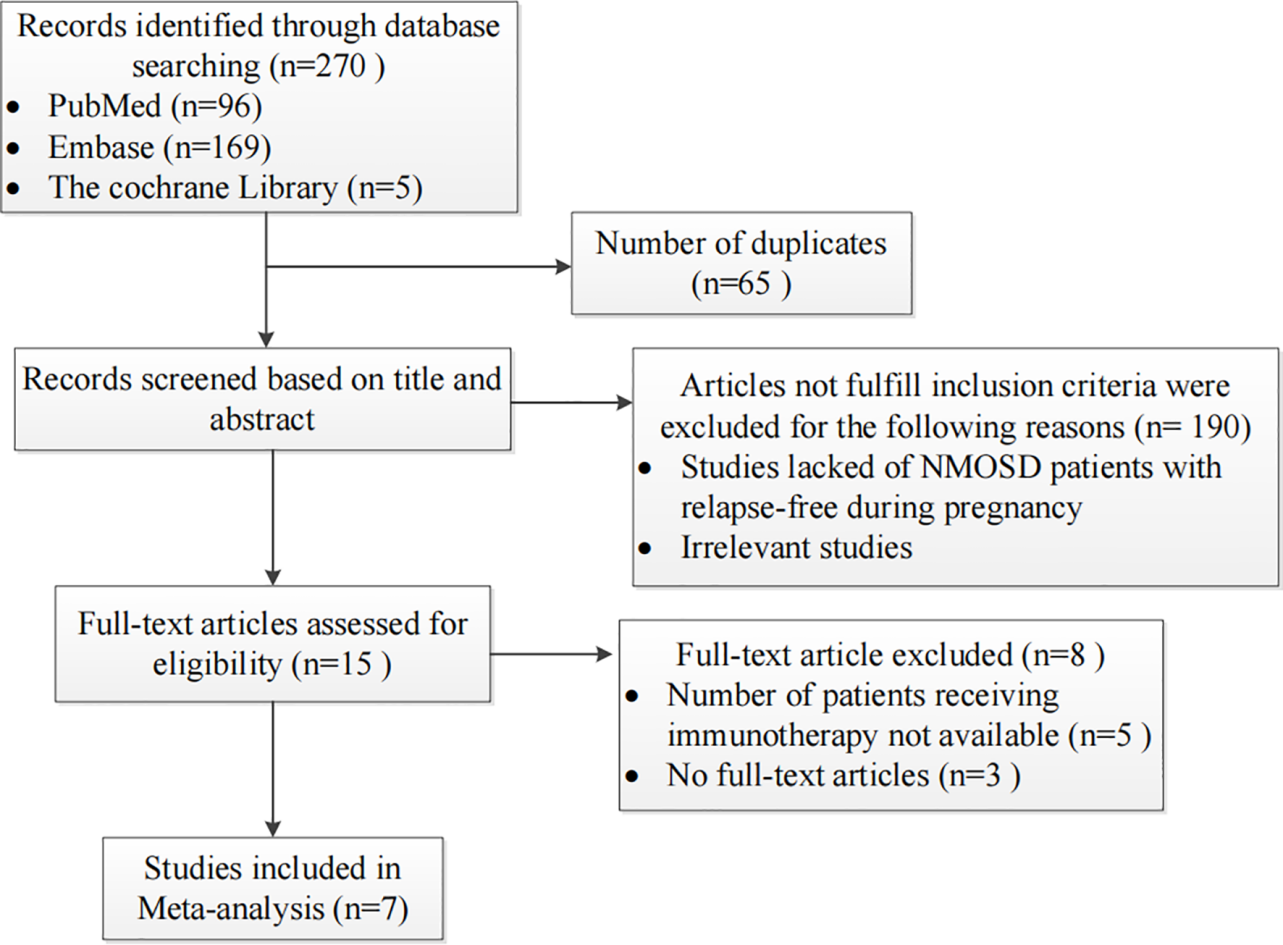
Flow chart presenting the process of study selection for the pregnancy-related neuromyelitis optica spectrum disorder (NMOSD) meta-analysis.

Two reviewers independently searched the PubMed, Embase, and Cochrane Library databases for articles published in English (without any temporal restrictions). A combination search of MeSH terms and keywords related to “pregnancy” and “neuromyelitis optica” was used in our meta-analysis. The studies were read thoroughly in order to evaluate the appropriateness of their inclusion in the meta-analysis. Cohort studies or case series comprising patients diagnosed with NMOSD prior to pregnancy or for the first-time during pregnancy were included. We excluded studies that enrolled fewer than five patients and those that did not examine the main variables of interest. From the 270 studies initially identified, 7 fulfilled the inclusion criteria and were included. The flowchart of the search strategy is shown in **Figure 1**. The RRs, miscarriage rates and occurrence rate of unhealthy newborns for NMOSD patients treated with or without IS during pregnancy were extracted from these studies. Two reviewers independently screened the titles, abstracts, and full texts; any differences in opinion were resolved *via* discussion and consensus.

#### 2.1.2 Statistical Analysis

The data extracted from the included studies were analyzed using Review Manager 5.4 software (RevMan Corp, Houston, TX, USA). Heterogeneity in RRs, miscarriage rates and occurrence rate of unhealthy newborns was determined using the I^2^ index, with an I^2^ value greater than or equal to 50% indicating moderate or high heterogeneity (19). When *p >*0.1 and I^2^ ≤ 50%, we used a fixed-effect model to conduct the meta-analysis; otherwise, a random-effects model was used. Statistical significance was set at *p <*0.05.

### 2.2 Retrospective Analysis

#### 2.2.1 Study Population

We collected data on clinical and diagnostic characteristics, therapy management, and pregnancy outcomes of pregnancy-related AQP4 positive NMOSD patients admitted to the Second Xiangya Hospital of Central South University between August 2000 and August 2020. All patients diagnosed with NMOSD met the diagnostic criteria established in 2015 (1), including those admitted before 2015. Cases lost to follow-up were excluded from the study. This study was approved by the Medical Ethics Committee of Second Xiangya Hospital of Central South University, and informed consent was obtained from all participants *via* telephone interview. This study had no adverse influence on the rights or welfare of patients. This study was conducted in accordance with the Declaration of Helsinki and with the utmost respect for patient privacy; we ensured the confidentiality of all patient information.

#### 2.2.2 Clinical Data Collection

We collected medical data for the 33 patients enrolled in this cohort, including age at conception, relapse rate, pregnancy outcomes, clinical manifestations, routine blood test results, serological analysis of autoantibodies, imaging features, Expanded Disability Status Scale (EDSS) scores, treatment management, and time interval between last relapse and conception. In NMOSD patients who received IS treatment, the blood test results used were those obtained at the beginning of the second trimester of pregnancy (since IS treatment started at the beginning of the second trimester of pregnancy), while in those with PRAs who did not receive IS treatment, these data were obtained at relapse, before IS treatment (intravenous methylprednisolone or intravenous immunoglobulin). Blood test results of NMOSD patients without PRAs who did not receive IS treatment were obtained at the beginning of the second trimester.

Blood sample were tested for analysis of auto-antibodies and NLR. AQP4-IgG was assessed using cell-based transfection immunofluorescence assay (CBA) in a laboratory of neuroimmunology (20). The NLR was calculated as the ratio of absolute neutrophil count to absolute lymphocyte count. This ratio is influenced by different physiological and medical conditions as well as by some medications. Some of the influencing factors include (1) pregnancy; (2) presence of autoimmune (including rheumatoid arthritis, SS, SLE, and inflammatory bowel disease), cardiometabolic (including diabetes mellitus, hypertension, and dyslipidemia) or liver diseases; malignancies; or hematologic conditions; (3) blood transfusions during the last four months, as well as use of antiplatelet medications (such as aspirin and clopidogrel); and (4) treatment with hormones, gamma-globulins, and immunosuppressive drugs in the past 6 months. For the analysis of NLR, we excluded the data of patients who met one or more of the above factors, except for autoimmune diseases and pregnancy. We also excluded those who lacked information on blood counts or had missing follow-up information in the medical records. Those with evidence of other infections were also excluded (13, 17, 21).

The EDSS scores were evaluated by two experienced neurologists to determine the severity of neurological deficits. EDSS scores were assessed at onset or relapse of NMOSD during pregnancy or within 1 year postpartum in patients with PRA, while it was assessed at the first trimester of the postpartum period in patients without PRA. At 1 year after delivery, all patients underwent EDSS evaluation again. Magnetic resonance imaging (MRI) results showing characteristic lesions in the optic nerve, spinal cord, and brain were defined as abnormal, while normal brain images and unspecific demyelinating brain lesions were defined as normal (1, 14).

Referring to previous research (22), the definitions used in the current study were as follows: (1) *informative pregnancies* were defined as all pregnancies occurring after the onset of NMOSD as well as all patients in whom the disease presented during the pregnancy or within 12 months postpartum; (2) *PRA* was defined as onset or relapse occurring during pregnancy or within 12 months after delivery or abortion; (3) *relapses* were defined as new or substantially worsened neurological symptoms lasting for at least 24 hours and a new or enhancing lesion on MRI so as to exclude pseudorelapses (23); (4) *pregnancy with IS treatment* was defined as a pregnancy in which the patient was on IS treatment (including corticosteroids, azathioprine [AZA], or rituximab) during at least 7 months of the pregnancy; (5) *pregnancy without IS treatment* was defined as a pregnancy in which the patient did not receive any IS treatment before or during pregnancy; and (6) *miscarriage* was defined as a spontaneous loss of intrauterine pregnancy during the first 24 weeks.

#### 2.2.3 Statistical Analysis

All statistical analyses were performed using Statistical Package for Social Sciences software (version 26.0; IBM Corp, Armonk, NY, USA). The Kolmogorov–Smirnov test was used to determine whether sample data were normally distributed. Continuous data conforming to a normal distribution were expressed as means ± standard deviations (SDs). When continuous data were normally distributed, we used Student’s t-tests to identify the differences between groups; otherwise, differences were examined using the Mann–Whitney U-test. The between-group differences in categorical variables were analyzed using Fisher’s exact tests. The predictive value of NLR for the prognosis of patients with NMOSD was analyzed using receiver-operating characteristic (ROC) curves, and the optimal cut-off point for continuous variables was determined based on the maximum Youden index (21). A P-value <0.05 was considered statistically significant.

## 3 Results

### 3.1 Pregnancy Outcomes

**Figure 2:**
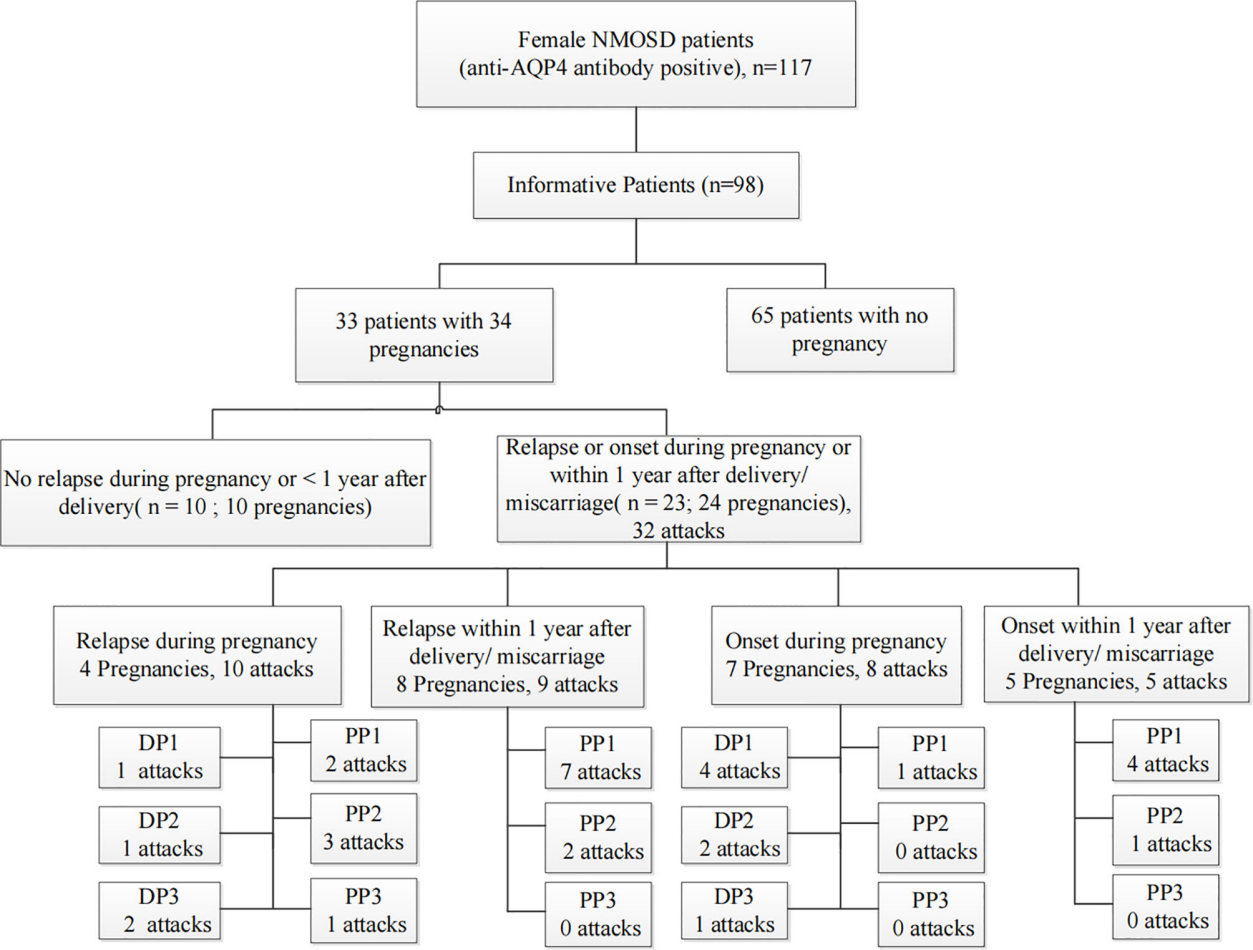
Summative statistics by study population. An informative pregnancy was defined as cases in which an attack occurred during pregnancy or within 1 year after delivery or abortion. DP, the period during pregnancy; DP1, during the first trimester of pregnancy; DP2, during the second trimester of pregnancy; DP3, during the third trimester of pregnancy; PP, within 1 year of the postpartum period; PP1, the first trimester of the postpartum period; PP2, the second trimester of the postpartum period; PP3, 7–12 months of the postpartum period.

We retrospectively screened 117 women with AQP4 antibody-positive NMOSD. Nineteen NMOSD patients were excluded because they were lost to follow-up. Among the 98 informative NMOSD patients, we enrolled 33 patients with informative pregnancies, which included 34 pregnancies in total. Of these, 23 patients (69.7%) had PRAs. **Figure 2** shows the clinical characteristics of the 23 patients who had at least one PRA. Attacks were most common during the first trimester after delivery/abortion (n=21, 65.6%).

### 3.2 Demographic and Clinical Characteristics

In total, 23 NMOSD patients with 24 informative pregnancies were included in this study. Of these, 12 (50%) were pregnant after NMOSD onset and 12 (50%) were diagnosed with NMOSD during pregnancy or within 1 year postpartum. The disease characteristics of NMOSD with informative pregnancies are presented in **Table 1**. The mean age at conception was 27.75 years, and the mean disease duration at the time of pregnancy was 1.5 months; these results were similar among the two groups. These 24 informative pregnancies included 13 term deliveries, 4 premature deliveries, 6 elective abortions, and 1 spontaneous miscarriage; further, 5 patients had preeclampsia. The reasons for elective abortions were NMOSD attacks resulting in serious neurological impairments during pregnancy (n=5) and concerns over medication side effects (n=1). Patients with single or multiple symptoms underwent disease confirmation using brain MRI-compatible lesions. NMOSD onset during pregnancy or within 1 year of the postpartum period was more likely to present with optic neuritis (*p*=0.012). The MRI-compatible lesions found in these 24 pregnancies included optic nerve lesions in 13 patients, myelitis lesions in 15 patients, lesions in the dorsal medulla/area postrema associated with area postrema syndrome in 4 patients, and other NMOSD-typical brain lesions in 9 patients. Fifteen patients had complications with other autoimmune diseases/antibodies, and extractable nuclear antigen positivity was the most common presentation in both groups (especially SS A/B-positive cases).

**Table 1:** Demographic and clinical characteristics of patients with pregnancy-related NMOSD attacks.

Pregnancies	Pregnancy after NMOSD onset, n=12	NMOSD onset during pregnancy or within 1 year of the postpartum period, n=12	*P*-value
Age at conception, years, mean (SD)	27.75 (5.04)	27.92 (4.74)	0.934
Mean disease duration at time of pregnancy, months, mean (SD)	1.5 (1.0)	2.1 (1.16)	0.198
Pregnancy outcome			
*Term delivery*	7	6	1.000
*Elective abortion*	1	5	0.155
*Premature delivery*	3	1	0.590
*Miscarriage*	1	0	1.000
*Preeclampsia*	2	3	1.000
Clinical syndromes, n	12	12	
*Myelitis and optic neuritis*	8	2	0.036^*^
*Optic neuritis and acute brainstem syndrome*	1	1	1.000
*Myelitis and area postrema syndrome*	1	0	1.000
*Optic neuritis and area postrema syndrome*	1	0	1.000
*Myelitis and acute brainstem syndrome*	0	2	0.478
*Myelitis and symptomatic cerebral syndrome with NMOSD-typical brain lesions*	0	1	1.000
*Symptomatic cerebral syndrome with NMOSD-typical brain lesions*	0	3	0.217
*Acute diencephalic clinical syndrome with NMOSD-typical brain lesions*	0	1	1.000
*Area postrema syndrome*	1	1	1.000
*Myelitis*	0	1	1.000
MRI Characteristic			
*Optic nerve lesion*	10	3	0.012^*^
*LETM*	9	6	0.400
*Dorsal medulla/area postrema lesions*	2	2	1.000
*Other NMOSD-typical brain lesions*	1	8	0.009^*^
Concomitant with autoimmune diseases/antibodies, n	10	5	0.089
*ANA*	4	2	0.640
*ENA*	9	5	0.214
*TPO- ab and TG- ab*	2	2	1.000

*p < 0.05. ANA, antinuclear antibody; ENA-ab, extractable nuclear antigen antibody; NMOSD, neuromyelitis optica spectrum disorder; TG-ab, thyroglobulin antibody; TPO-ab, thyroid peroxidase antibody; LETM, Longitudinal extensive transverse myelitis; Mean disease duration at time of pregnancy: the time from the symptom onset to alleviation.

### 3.3 Effect of Clinical Indicators on the Severity of Neurological Dysfunction

In order to evaluate the effects of the clinical indicators on the severity of neurological impairment at disease onset or relapse in patients with pregnancy-related NMOSD attacks, we grouped the pregnancy-related NMOSD patients into mild-to-moderate and severe groups according to their EDSS scores (≤3 or >3) as previously described (14). **Table 2** shows the comparisons of demographic and clinical features according to EDSS scores during pregnancy based on NMOSD onset or relapse. Compared with patients in the mild-to-moderate group, those in the severe group were more likely to have MRI abnormalities in the spinal cord (*p*<0.001) or present with combined myelitis and optic neuritis symptoms (*p*=0.019). Besides, concomitant antibodies were more commonly seen in the severe group (*p*=0.004).

**Table 2:** Clinical characteristics of patients with pregnancy-related NMOSD attack according to the EDSS score at NMOSD onset or relapse.

Clinical Characteristics	EDSS (≤3), n = 7	EDSS (>3), n = 17	*P*-value
Age at conception, years, mean (SD)	29.57 (4.39)	27.18 (4.84)	0.271
AQP4 IgG titers			1.000
≤1:32	4	8	
>1:32	3	9	
Clinical manifestations, n			
*Optic neuritis and acute brainstem syndrome*	2	0	0.076
*Myelitis and optic neuritis*	0	10	0.019^*^
*Myelitis and symptomatic cerebral syndrome*	0	1	1.000
*Optic neuritis and area postrema syndrome*	1	0	0.292
*Myelitis and area postrema syndrome*	0	1	1.000
*Myelitis and acute brainstem syndrome*	0	2	1.000
*Myelitis*	0	1	1.000
*Area postrema syndrome*	2	0	0.076
*Symptomatic cerebral syndrome*	1	2	1.000
*Acute diencephalic clinical syndrome*	1	0	0.292
MRI Characteristic			
*Optic nerve lesion*	3	10	0.659
*LETM*	0	15	<0.001^*^
*Dorsal medulla/area postrema lesions*	3	1	0.059^*^
*Other NMOSD-typical brain lesions*	4	5	0.356
Concomitant with autoimmune diseases/antibodies, n	1	14	0.004^*^

*p < 0.05. NMOSD, neuromyelitis optica spectrum disorder; LETM, Longitudinal extensive transverse myelitis.

### 3.4 IS Treatment During Pregnancy

In our study, all the patients suspended IS treatment six months before pregnancy. Among these, 14 patients were relapse free during the first trimester and started to receive IS treatment at the beginning of the second trimester of pregnancy (to avoid adverse effects of the drugs on the fetus during early pregnancy). Among these 14, 6 patients were treated with 10 mg prednisone, 5 were treated with 40 mg prednisone, 1 received two rituximab injections during the second trimester of pregnancy and one rituximab injection every six months after delivery, and 2 were treated with AZA (at 100 mg) along with 20 mg of prednisone. Ten patients were relapse-free during pregnancy, eight accepted IS treatment, and two received no drug treatment (these patients were diagnosed with optic neuritis before pregnancy). Of the 8 patients who received IS treatment, the pregnancy outcomes included five term deliveries, two premature deliveries, and one elective abortion (due to concerns about the side effects of AZA). Additionally, 6 patients were treated with 10 mg prednisone only; all of these patients had a relapse during pregnancy. Among these 6 patients, the pregnancy outcomes included 1 term delivery, 3 premature deliveries, and 2 elective abortions due to severe neurological dysfunction during pregnancy (n=2).

**Figure 3:**
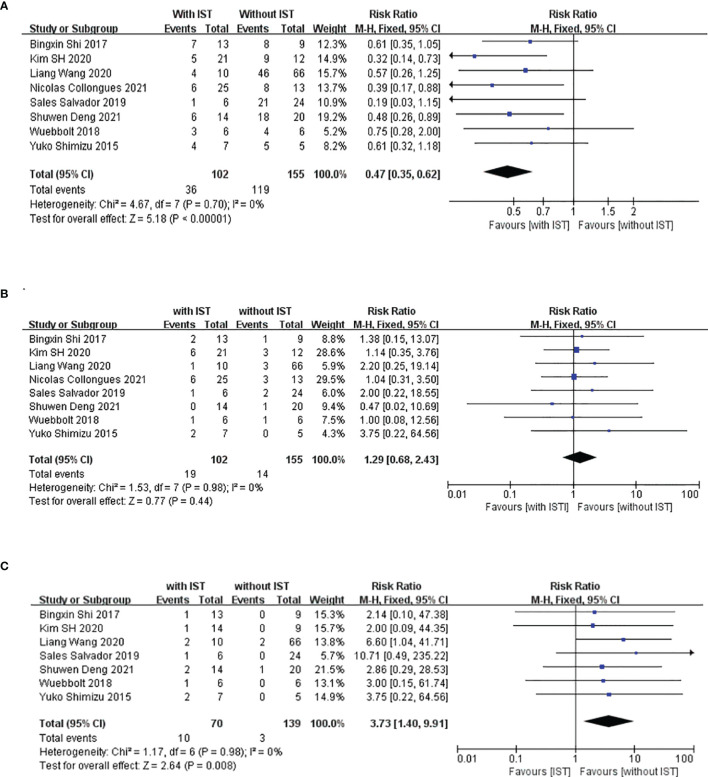
Meta-analysis of immune-suppression treatment during pregnancy and relapse rate (RR), incidence of unhealthy neonates, and miscarriage. This figure presents a meta-analysis of 8 studies (including our study) of the association between maintenance immunosuppression treatment during pregnancy and RR **(A)**, miscarriage rate **(B)** and incidence of unhealthy neonates **(C)**.

We compared the RRs of patients treated with and without IS treatment during pregnancy; retrospective studies have been conducted in this regard in eight other study populations, including ours (5, 22, 24–28). The main characteristics of these studies are summarized in **Table 3**. These studies were all retrospective, and 167 patients with 223 PRAs from seven studies were included in the meta-analysis. **Figure 3A** shows a forest plot of the mean differences in the RR ratio before and after IS therapy. Heterogeneity analysis showed results of I=0% and *p*=0.70 within the Q test, suggesting that there was no heterogeneity among the studies selected for our analysis. Therefore, we decided to use the fixed-effects model to calculate the combined effect quantity. The combined effect RR for the 8 included studies was 0.47 (0.35-0.62; z=5.18, *p*<0.001). This result indicates that maintenance of IS treatment during pregnancy can significantly reduce the RR of NMOSD. The RR for those receiving IS treatment was 0.47 times that of patients not receiving treatment during pregnancy. Because only eight studies were included, a funnel plot was not constructed.

**Table 3:** Clinical characteristics of 167 patients with 223 pregnancy-related neuromyelitis optica spectrum disorder attacks from 7 studies included in the meta-analysis.

Reference (year)	Type of research	Patient No.	Pregnancies No.	Pregnancy Outcome	AQP4 IgG(+) during pregnancy No.
**Kim et al. (** 5 **)**	Retrospective	29	33	Kim et al. Term delivery and Premature delivery (24) Miscarriage (6)	32
**Collongues et al. (** 22 **)**	Retrospective	28	38	Collongues et al. Term delivery and Premature delivery (28) Miscarriage (9)	38
**Wang et al. (** 24 **)**	Retrospective	60	76	Wang et al. Term delivery and Premature delivery (43) Miscarriage (4)	50
**Shi et al. (** 25 **)**	Retrospective	16	22	Salvador et al. Term delivery (13) Premature delivery (20) Miscarriage (3)	18
**Salvador et al. (** 27 **)**	Retrospective	19	30	Term delivery (1) Premature delivery (5) Miscarriage (3)	30
**Wuebbolt et al. (** 28 **)**	Retrospective	4	12	Term delivery (5) Premature delivery (5) Miscarriage (2)	9
**Shimizu et al. (** 26 **)**	Retrospective	11	12	Term delivery (9) Premature delivery (2) Miscarriage (2)	12

In addition, we also compared the miscarriage rates and occurrence rate of unhealthy newborns between patients who underwent and did not undergo IS treatment during pregnancy to explore the safety of IS treatment in the abovementioned eight populations. **Figure 3B** shows a forest plot of the mean differences in the miscarriage rate before and after IS therapy. The combined effect of the miscarriage rate for the 8 included studies was 1.29 (95%CI=0.68-2.43; z=0.77, *p*=0.44). This result indicates that maintenance of IS treatment during pregnancy had no effect on the miscarriage rate for NMOSD. However, the unhealthy newborn proportions for those receiving IS treatment was 3.73 times that of patients not receiving IS treatment during pregnancy (95%CI=1.40–9.91; z=2.64, *p*=0.008) (**Figure 3C**), which suggests that IS treatment during pregnancy may have an adverse effect on the health of the neonates. Among the 209 neonates included in this meta-analysis, 13 were unhealthy, with diagnoses of low birth weight (n=7), splenomegaly (n=1), thrombocytopenia (n=1), undeveloped external ear (n=1), dacrocyte obstruction and scoliosis (n=1), congenital fusion of the syndactyly, and cerebral ischemia(n=1).

### 3.5 Variables as Predictor of Pregnancy Related Attack in NMOSD

We compared demographic and clinical factors between NMOSD patients with and without PRAs (**Table 4**). The EDSS score at the PRA phase or up to 1 year after pregnancy did not differ between the two groups. However, the EDSS score increased significantly at 1 year after delivery/abortion in pregnancies with PRAs compared with the EDSS score at relapse during pregnancy (*p*=0.039).

**Table 4:** Comparison of clinical characteristics and the outcomes between NMOSD patients with and without pregnancy-related NMOSD attack.

Clinical Characteristics	Pregnancies after NMOSD onset with PRA, n=24	Pregnancies without pregnancy-related attack, n=10	*P*-value
Age at conception, years, mean (SD)	27.88 (4.75)	28.02 (4.76)	0.945
Pregnancy outcome			
*Term delivery*	13	7	0.467
*Elective abortion*	6	1	0.644
*Premature delivery*	4	2	1.000
*Miscarriage*	1	0	1.000
*Preeclampsia*	5	1	0.794
NLR at conception or within 1 year of the postpartum period (prior to IS treatment), mean (SD)	7.85 (3.98)	3.98 (1.08)	<0.001^*^
EDSS score during conception or within 1 year of the postpartum period, mean (SD)	4.84 (1.94)	4.70 (1.23)	0.826
EDSS score at 1 year after delivery, mean (SD)	5.95 (1.61)	4.95 (1.21)	0.086
Unhealthy newborn	1	2	0.201
Concomitant with autoimmune diseases/antibodies, n	15	3	0.134

*p < 0.05. NLR, neutrophil-to-lymphocyte ratio; NMOSD, neuromyelitis optica spectrum disorder; EDSS, Expanded Disability Status Scale.

**Figure 4:**
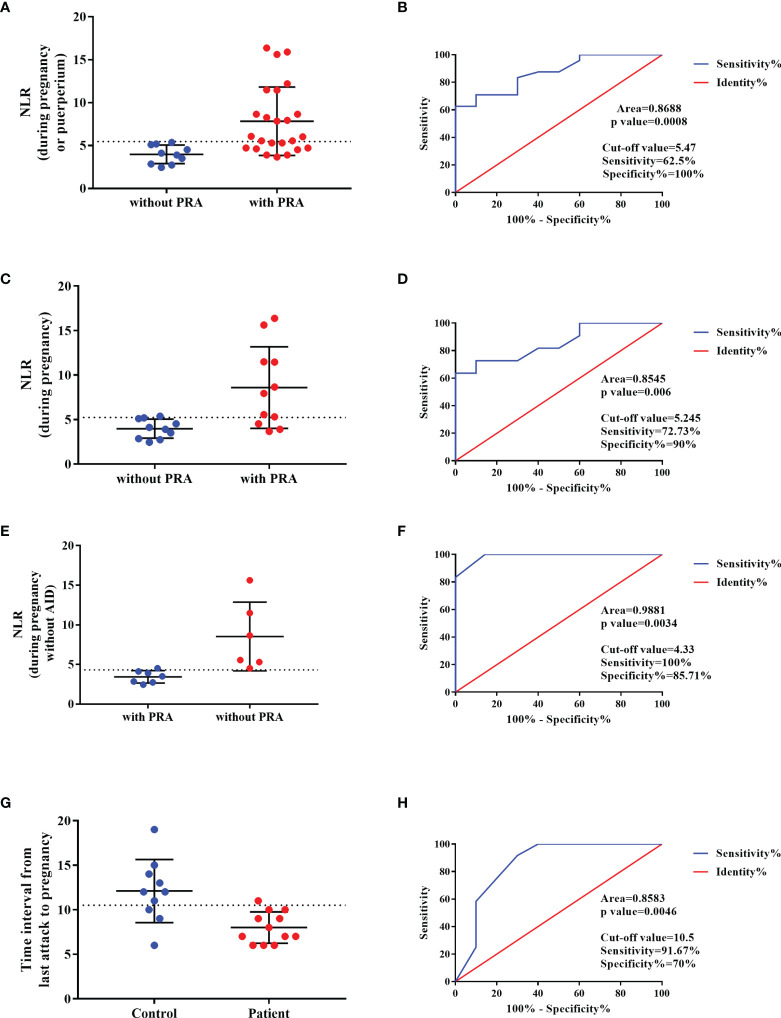
Predictor of the NMOSD relapse during pregnancy. **(A, C, E)** NLR value in NMOSD with pregnancy-related attacks (PRAs) compared to those with NMOSD who were PRA-free, and the ROC curve for this is presented in **(B, D, F)**, respectively. **(G)** The time interval between last relapse and conception in NMOSD patients with PRA compared with NMOSD patients without PRA and the ROC curve was built in **(H)**.

Those with NMOSD with PRAs exhibited a higher NLR at the beginning of the second trimester or at relapse (prior to methylprednisolone therapy or PLEX treatment; *p*<0.001) as compared to those with NMOSD who were PRA-free (**Figure 4A**). As mentioned before, autoimmune diseases/antibodies and pregnancy status can also influence NLR; therefore, we conducted a subgroup analysis to avoid the influence of the pregnancy and autoimmune antibody status. We first grouped NMOSD patients with PRA into two groups: one group of subjects who had an attack during pregnancy, and a second group comprised of subjects who had an attack within the first year of the postpartum period; we compared the NLR values during pregnancy in NMOSD patients with and without PRAs (**Figure 4C**). Next, we excluded the NLR value in patients with concomitant autoimmune antibodies and performed a comparison to address the confounders of pregnancy status and autoimmune antibody (**Figure 4E**). As can be seen in **Figures 4C, E**, a higher NLR was seen in those with NMOSD with PRAs, and the ROC curve for this is presented in **Figures 4B, D, F**.

The time interval between last relapse (with typical NMOSD-MRI lesions) and conception was shorter in NMOSD patients with PRA compared with NMOSD patients without PRA (**Figure 4G**). Based on the ROC curve, the optimal cut-off values of the time interval for the prediction of the NMOSD relapse during pregnancy was 10.5 months (**Figure 4H**).

## 4 Discussion

NMOSD is primarily mediated by humoral immunity, and pregnancy plays a regulatory role in the development of NMOSD (25). Pregnancy induces significant changes in the hormonal and immunological environment. The fetoplacental unit synthesizes Th2 cytokines that induce the downregulation of maternal Th1 cytokines, thereby mediating cellular immunity, increasing humoral immunity, and helping maintain a successful pregnancy (30). However, an enhanced generation of autoantibodies is stimulated by increased Th2-mediated immune responses and elevated maternal humoral immunity during pregnancy (31). We analyzed the pregnancy-related characteristics of patients with AQP4-positive NMOSD and found that some cases had disease onset during pregnancy. Among the 23 patients with PRAs, 12 were first diagnosed with NMOSD during pregnancy or within 1 year after delivery/abortion. The onset of NMOSD during pregnancy may result from immunological changes occurring during pregnancy, which may stimulate the production of AQP4-ab. AQP4 is expressed in the human placenta during gestation and plays a regulatory role in the maternal–fetal fluid exchange between placental cells and fetal capillaries in healthy mothers (32, 33). We found increased levels of AQP4 in the placenta and fetal membrane in a patient with acute disseminated encephalomyelitis onset in pregnancy as compared to that in a healthy control; this may be due to the activation of the HMGB1/TLR4/Nf-kB/IL-6 pathway (34). Therefore, we speculate that the increased levels of AQP4 on the placenta were mistakenly identified as “foreign proteins” due to the alteration of the immunological state during pregnancy, leading to the occurrence of autoimmune reactions and stimulating production of AQP4-ab in the serum. However, the miscarriage rate in our study was low (1/3), which is inconsistent with previous findings that NMOSD induces high miscarriage rates (5, 35). Further studies are needed to determine whether anti-AQP4 IgG-mediated pathogenesis increases the miscarriage rate. Additionally, we observed that the attacks commonly occurred in the first trimester of the postpartum period after delivery/abortion (65.6%), which is in accordance with the results of a previous cohort study of pregnancy-related NMOSD (24).

Optic neuritis and myelitis are the most common manifestations of pregnancy-related NMOSD. However, it was interesting to find a slight discrepancy in MRI characteristics between the pregnancy-related NMOSD subgroups. Lesions in the optic nerve are more common in patients who become pregnant after NMOSD onset, whereas patients diagnosed with NMOSD during pregnancy or within 1 year after delivery/abortion more commonly presented with acute encephalic lesions. To our knowledge, this discrepancy was first observed in our study, although these findings require larger cohorts for confirmation.

Attack during pregnancy significantly increased disability in NMOSD patients at 1 year after pregnancy (12). The EDSS score increased significantly at 1 year after delivery or abortion in pregnancies with PRAs, which was also demonstrated in a study by Shi et al. (25). In addition, pregnancy-related NMOSD concomitant with autoimmune diseases/antibodies increased the severity of neurological injury. In our study, the most common autoimmune disease coexisting with NMOSD was SS with SS-A and SS-B positivity. The existence of the SS-A antibody may represent a state of enhanced autoimmune inflammatory response and lead to more severe inflammatory damage. A similar result was found in a study conducted by Akaishi et al, showing that the EDSS score for the acute phase in NMOSD was significantly higher in patients with comorbid SS than in those without comorbid SS (36). Gupta et al. demonstrated that the severity and relapse rates for SS were exacerbated during the period after delivery (37).

Pregnancy without PRAs is associated with IS treatment. We reviewed eight populations (including those from our study) and performed a meta-analysis the results of which demonstrated that IS treatment can reduce the risk of relapse during pregnancy as well as in the postpartum period. Collongues et al. observed a tendency among patients who continued their treatment during pregnancy to have fewer relapses as compared to those who interrupted their treatment before pregnancy (22). Moreover, Wang et al. suggested that maintenance therapy with appropriate immunosuppression or a sufficient dose of oral steroids should be administered during pregnancy as well as during the postpartum period (24).

The maintenance of IS treatment during pregnancy needs to be considered on an individual basis. Previous studies have stated that it is safe to use low-dose prednisone during pregnancy, as the estimated fetal level is only 10% of the maternal level (25, 38). However, chronic use of corticosteroids during pregnancy may result in premature delivery; 2 of 13 patients who received prednisone in our study had premature deliveries, and both of them delivered a baby with low birth weight. Previous studies revealed that daily use of low-dose prednisone does not affect fetal health; however, this type of use does not reduce the RR (25–27). Similar results were found in our study since six patients treated only with 20 mg prednisone had a relapse during pregnancy. Therefore, the literature to date indicates that the dosage of prednisone should be upregulated during single drug maintenance; more studies are needed to identify the specific dose. Although AZA may be a safe therapy for patients with NMOSD occurring during pregnancy (39), many patients in our cohort refused to use AZA during pregnancy because of concerns about the adverse effects of this drug. In relation to this, monoclonal antibodies are increasingly used in pregnancy (40, 41). In our cohort, only one patient, who received rituximab therapy, was relapse-free during pregnancy and delivered a healthy baby. The effectiveness and safety of rituximab for NMSOD, administered in pregnancy, has been rarely reported in cohort studies (29). A cohort study conducted in Germany demonstrated that anti-CD20 mAbs can effectively control progression and relapse in women with NMOSD during pregnancy or after delivery or abortion; moreover, it is safe to use CD20 mAbs within 6 months prior to pregnancy (42). Until now, limited data has been reported on the effectiveness and safety of IS treatment. The meta-analysis conducted in this study showed that the maintenance of IS treatment during pregnancy had no adverse effect on the miscarriage rate among those with NMOSD, while the occurrence rate of unhealthy newborns was elevated. Low birth weight was most common presentation among the unhealthy newborns, based on summarized data from the included studies, despite the fact that this factor has little life-threatening effect for newborns. However, administration of IS treatment during pregnancy still needs careful consideration.

At present, the relevance of the NLR in the recurrence or onset of NMOSD during pregnancy is unclear. Previous studies have observed a correlation between the NLR and RR in NMOSD. Zhou et al. demonstrated that the NLR is an independent risk factor for the severity of neurological dysfunction in patients at the first attack of NMOSD (14). Contentti et al. suggested that the NLR does not appear to act as an independent predictor of worse outcomes and that the NLR may be quite limited as a biomarker of disease activity (13). Therefore, the predictive role of the NLR as an independent predictor of worse outcomes is controversial. To our knowledge, our study is the first to analyze the relationship between the NLR and pregnancy-related NMOSD. We found that pregnancy with a PRA was associated with a higher NLR at relapse during pregnancy and that the value increased significantly compared with that during the remission period. According to our results, the NLR may be an effective predictor of PRAs. In addition, it would be crucial to determine the disease activity prior to pregnancy (the time interval between last relapse and conception) since it can be a predictor of relapse attack during pregnancy.

The primary limitations of our study include its retrospective nature and small sample size. Both the generalizability and robustness of our findings may be affected by these limitations. Even though increased NLR may be predictor for PRAs in NMOSD, it is influenced by different physiological and medical conditions as well as pregnancy itself. Further prospective studies with larger sample sizes are needed to clarify our findings. Besides, the EDSS score retrospectively evaluated from the patient chart may influence the result in this study. In addition, relatively few studies were included in our meta-analysis due to the sparsity of the literature; the funnel plot to exclude publication bias was, therefore, not constructed.

Our study demonstrates that pregnancy-related NMOSD attacks occurred mostly in the first trimester after delivery/abortion. Moreover, increased NLR and short time interval between last relapse and conception may be a predictor for PRAs in NMOSD. In addition, maintenance of IS therapy during pregnancy and after delivery or abortion can reduce the risk of relapse; however, it can also increase the incidence of unhealthy newborns. Therefore, administration of IS treatment during pregnancy to prevent relapse should be done considering the dosage of drugs and after evaluating the risks of adverse effects to the fetus.

## Data Availability Statement

The raw data supporting the conclusions of this article will be made available by the authors, without undue reservation.

## Ethics Statement

This study was approved by the Medical Ethics Committee of Second Xiangya Hospital of Central South University, and informed consent was obtained from all participants via telephone interview. This study had no adverse influence on the rights or welfare of patients. This study was conducted in accordance with the Declaration of Helsinki and with the utmost respect for patient privacy; we ensured the confidentiality of all patient information.

## Author Contributions

SD drafted the manuscript. QL and SD carried out the literature review. WL contributed to and finalized the draft. All authors contributed to the article and approved the submitted version.

## Funding

This work was funded by the Natural Science Foundation of Hunan Province (#2020JJ4798).

## Conflict of Interest

The authors declare that the research was conducted in the absence of any commercial or financial relationships that could be construed as a potential conflict of interest.

## Publisher’s Note

All claims expressed in this article are solely those of the authors and do not necessarily represent those of their affiliated organizations, or those of the publisher, the editors and the reviewers. Any product that may be evaluated in this article, or claim that may be made by its manufacturer, is not guaranteed or endorsed by the publisher.
